# Antimony–Platinum Modulated Contact Enabling Majority Carrier Polarity Selection on a Monolayer Tungsten Diselenide Channel

**DOI:** 10.1021/acs.nanolett.4c01436

**Published:** 2024-07-09

**Authors:** Yu-Tung Lin, Ching-Hao Hsu, Ang-Sheng Chou, Zi-Yun Fong, Chih-Piao Chuu, Shu-Jui Chang, Yu-Wei Hsu, Sui-An Chou, San Lin Liew, Ting-Ying Chiu, Fa-Rong Hou, I-Chih Ni, Duen-Huei Vincent Hou, Chao-Ching Cheng, Iuliana P. Radu, Chih-I Wu

**Affiliations:** § Graduate Institute of Photonics and Optoelectronics, National Taiwan University, Taipei 106, Taiwan; ¶ Corporate Research, Taiwan Semiconductor Manufacturing Company, Hsinchu 30091, Taiwan; † Quality & Reliability, Taiwan Semiconductor Manufacturing Company, Hsinchu 30091, Taiwan

**Keywords:** two-dimensional materials, tungsten diselenide, polarity transition, field-effect transistor, contact modulation, interface analysis, photoemission spectroscopy

## Abstract

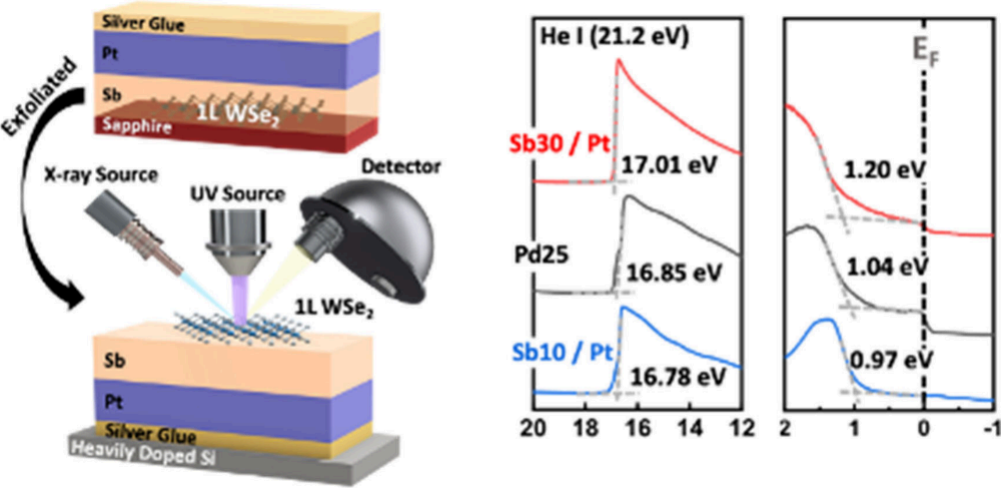

We develop a novel metal contact approach using an antimony (Sb)–platinum (Pt) bilayer to mitigate Fermi-level pinning in 2D transition metal dichalcogenide channels. This strategy allows for control over the transport polarity in monolayer WSe_2_ devices. By adjustment of the Sb interfacial layer thickness from 10 to 30 nm, the effective work function of the contact/WSe_2_ interface can be tuned from 4.42 eV (p-type) to 4.19 eV (n-type), enabling selectable n-/p-FET operation in enhancement mode. The shift in effective work function is linked to Sb–Se bond formation and an emerging n-doping effect. This work demonstrates high-performance n- and p-FETs with a single WSe_2_ channel through Sb–Pt contact modulation. After oxide encapsulation, the maximum current density at |V_D_| = 1 V reaches 170 μA/μm for p-FET and 165 μA/μm for n-FET. This approach shows promise for cost-effective CMOS transistor applications using a single channel material and metal contact scheme.

Nearly atomically thin two-dimensional (2D) transition-metal dichalcogenides (TMDs) are promising channel materials for future scaled logic electronics,^1−6^ based on their excellent electrostatic control in monolayer (1L) thickness.^7^ In general, TMDs are composed of transition metal atoms, such as tungsten (W) or molybdenum (Mo), that are sandwiched between two layers of chalcogen atoms, such as sulfur (S), selenium (Se), or tellurium (Te), by strong intralayer covalent bonding. Tungsten diselenide (WSe_2_) has gained significant attention because of the direct band gap of ∼1.65–1.75 eV^8,9^ and high electron and hole mobilities,^10−14^ making it promising for complementary metal oxide semiconductor (CMOS) applications.^15−18^ However, the position of the Fermi-level pinning (FLP), arising from the metal-induced gap states (MIGS) and interface traps, has been identified to be close to midgap at the metal–WSe_2_ interface.^19,20^ This FLP issue in metal contacts causes high Schottky barriers (0.5–0.6 eV) for both electron and hole transport in WSe_2_ transistors.^21^ Among common metals studied in the literature,^22−24^ palladium (Pd) contact was explored as a proper p-contact on WSe_2_ because of its high work function, good oxidative stability, and better adhesion to 2D materials.

Using semimetal contacts is considered as another potential solution in 2D transistors to minimize MIGS in the vicinity of the Fermi level (E_F_). Monolayer molybdenum disulfide (MoS_2_) n-FETs were demonstrated to exhibit a low contact resistance down to a few hundred Ω·μm by using bismuth (Bi) or antimony (Sb) as the contact metals.^25−28^ These semimetals, in general having low work functions, are expected to enhance the electron conduction in 1L-WSe_2_ but are nonfavorable for hole conduction due to the improper band alignment.

This work introduces a contact modulation technique utilizing a simple Sb–Pt metal stack to mitigate the FLP issue in 1L-WSe_2_, enabling us to effectively adjust the work function at the contact interface. By employing this approach, high performance n-FET and p-FET of the 1L-WSe_2_ channel are successfully demonstrated, outperforming the Pd contacted WSe_2_ devices. Several physical characterizations are conducted to understand the underlying mechanism occurring at the WSe_2_ contact interface: (i) cross-sectional scanning transmission electron microscopy (STEM) imaging and corresponding energy-dispersive X-ray spectroscopy (EDS) mapping are adopted to validate the actual contact profile on WSe_2_; (ii) ultraviolet photoelectron spectroscopy (UPS) is used to characterize the effective metal work function after contacting 1L-WSe_2_; and (iii) X-ray photoelectron spectroscopy (XPS) is performed to analyze the chemical bonding formed at the metal–WSe_2_ interface. These physical inspections in combination with the density functional theory (DFT) calculation can correlate to the changes in electrical performance.

In experiments the back-gated device structure is fabricated, as the scheme shown in Figure S1a. The 1L-WSe_2_ film is precisely transferred onto a Si/SiN_*x*_ substrate with a thickness of 100 nm. An annealing process at 200 °C is carried out to eliminate polymer contaminants from the transfer process. Then the source/drain (S/D) photoresist pattern is formed using helium ion-beam lithography (HIBL), followed by depositing different thicknesses of the Sb layer (10–30 nm) and a 12 nm Pt capping layer. Contact metals were deposited through an electron-beam evaporator with a rate of 0.6 Å/s at the pressure of 1 × 10^–7^ Torr. The S/D contact of the Sb–Pt bilayer is obtained through the standard metal lift-off procedure. The final step is to define the active area by using O_2_ plasma etching, followed by immersion in dilute NH_4_OH (NH_4_OH:H_2_O = 1:4) to eliminate the remaining WO_*x*_ residue. The actual device of the 1L-WSe_2_ channel with the Sb–Pt metal contacts is presented in the optical microscopy (OM) image in Figure S1b. The scanning electron microscopy (SEM) image (Figure S1c) showcases that the channel length and width are 100 nm and 2 μm, respectively. Besides, the WSe_2_ step height is characterized by atomic force microscopy (AFM) analysis (Figure S1d), showing a thickness of approximately 0.9 nm and smooth surface topology, indicative of the monolayer characteristics and good transfer procedure for the WSe_2_ transferred film.

Figure 1a displays the typical transfer characteristics (I_D_–V_G_) of the Sb–Pt contacted WSe_2_ FETs with varying Sb thicknesses, along with the devices with Pd contact for comparison. As compared to the Pd-contacted device, the device with the 10 nm-Sb/Pt contact shows a higher on-state current in the p-branch, while the 30 nm-Sb/Pt contacted device shows a higher on-state current in the n-branch. In other words, the WSe_2_ device in contact with a thinner layer of Sb exhibits notable p-FET behavior, whereas a device with a thicker Sb layer displays more n-FET characteristics. We thus compare the difference in on-current under the same overdrive voltage (V_ov_), as in the line chart plotted in Figure 1b. In the n-branch region, on-current does increase as the Sb thickness increases. The 30 nm-Sb/Pt contacted device shows the on-current up to 42 μA/μm, 10 times higher than the reference Pd contact. In the p-branch region, 10 nm-Sb/Pt contacted device shows a relatively large on-current of 12 μA/μm, still 3 times higher than the Pd contacted device. We speculate that the transition of the majority carrier polarity in ambipolar WSe_2_ transistors is linked to the interfacial reaction and/or alteration in the band alignment between WSe_2_ and the Sb–Pt contact.

**Figure 1:**
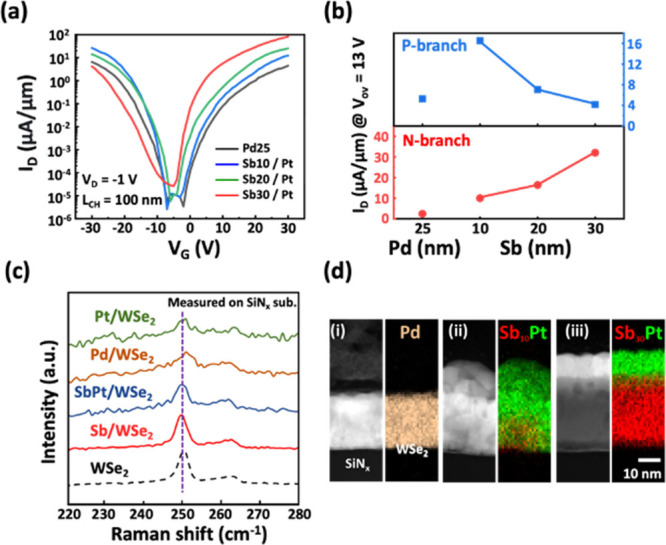
Characteristics of 1L-WSe_2_ FETs on the Si/SiN_*x*_ substrate with Sb/Pt modulated contact. (a) Transfer characteristics with different Sb thicknesses under Pt contacts compared to typical Pd contact. (b) Summary of n-branch and p-branch on-currents at fixed voltage overdrive as extracted from data in Figure 1a. (c) Raman spectroscopy of pristine 1L-WSe_2_ (black dotted curve) compared to samples capped with 1.5 nm of Pt (green curve), Pd (brown curve), Sb (red curve), and Sb/Pt (blue curve). (d) Cross-sectional STEM images and EDS mapping of 1L-WSe_2_ capped with (i) 25 nm Pd, (ii) 10 nm Sb and 12 nm Pt, and (iii) 30 nm Sb and 12 nm Pt.

Therefore, the damage risk from the deposition of high melting point metals on the WSe_2_ material is evaluated by Raman measurement. Ultrathin thickness of the metal is deposited in order not to obstruct the incident light or attenuate the local light field on WSe_2_ during Raman measurement. As shown in Figure 1c, several metal schemes are tested to examine the change in the quality of 1L-WSe_2_. The deposition of 1.5 nm Pd and Pt metals is found to broaden the characteristic peak of the as-deposited WSe_2_ with a reduced intensity. The WSe_2_ spectrum remains the same upon the deposition of Sb, which further helps to mitigate the intensity drop in the following Pt deposition. We infer that high work-function metals like Pd and Pt do induce some degree of perturbation in the WSe_2_ lattice.^29,30^ This disturbance can be manifested as the introduction of defects^31,32^ or as interfacial chemical bonding, thereby resulting in the defect-induced gap states (DIGS) or MIGS, *i.e*., FLP effect. In other words, the semimetallic Sb layer causes less disruption to the WSe_2_ lattice. Therefore, the contact interface profiles are compared by STEM images and EDS mappings, as presented in Figure 1d. In the case of 10 nm-Sb/Pt, only a thin Sb layer remains at the interface along with the Sb–Pt intermixing, which could be attributed to the resublimation of Sb during Pt evaporation. With increasing Sb layer to 30 nm, metal contact to the WSe_2_ interface is dominated by semimetallic Sb because of negligible intermixing. The phenomenon of Sb–Pt intermixing is deduced to change the effective work function in contact and consequently determine the device performance. This finding would be verified by physical inspections later.

For statistical analysis in Sb–Pt contacted devices, we conducted measurements across more than 100 devices in the I_D_–V_G_ characteristics (Figure 2a). The devices with 10 nm-Sb/Pt and 30 nm-Sb/Pt contacts exhibit p-FET and n-FET dominant behavior, respectively, which achieves maximum on-current of 27 and 95 μA/μm, respectively, at a gate voltage of ±30 V, as illustrated in Figure 2b. Moreover, the modulation of Sb–Pt contacts also enables enhancement mode operation in devices. The values of threshold voltage (V_th_) are −14 V (p-FET) and 2 V (n-FET) when using the 10 nm-Sb/Pt and 30 nm-Sb/Pt contacts, respectively (see Figure 2c). It is found that the p- and n-branch V_th_ in the 30 nm-Sb contact show a negative V_th_ shift as compared to the thinner Sb one. We thus need to understand the dominant factor in V_th_ of the scaled devices. As the resistance partition of WSe_2_ devices in Figure 2d, the channel resistance in n-/p-FETs is lower than the contact resistance, indicative of contact-controlled V_th_ behavior. As a result, the V_th_ shift in both current branches is dominated by the contact. Overall, with increasing Sb thickness in contact, we observe the carrier polarity change to n-type in devices along with a negative V_th_ shift. Less Sb–Pt intermixing with minor damage in WSe_2_ is also characterized. These structural changes in contacts would cause the interfacial reaction to induce the charge transfer and/or n-doping effect. As a result, the energy band alignment at the contact–WSe_2_ interface could be modified.

**Figure 2:**
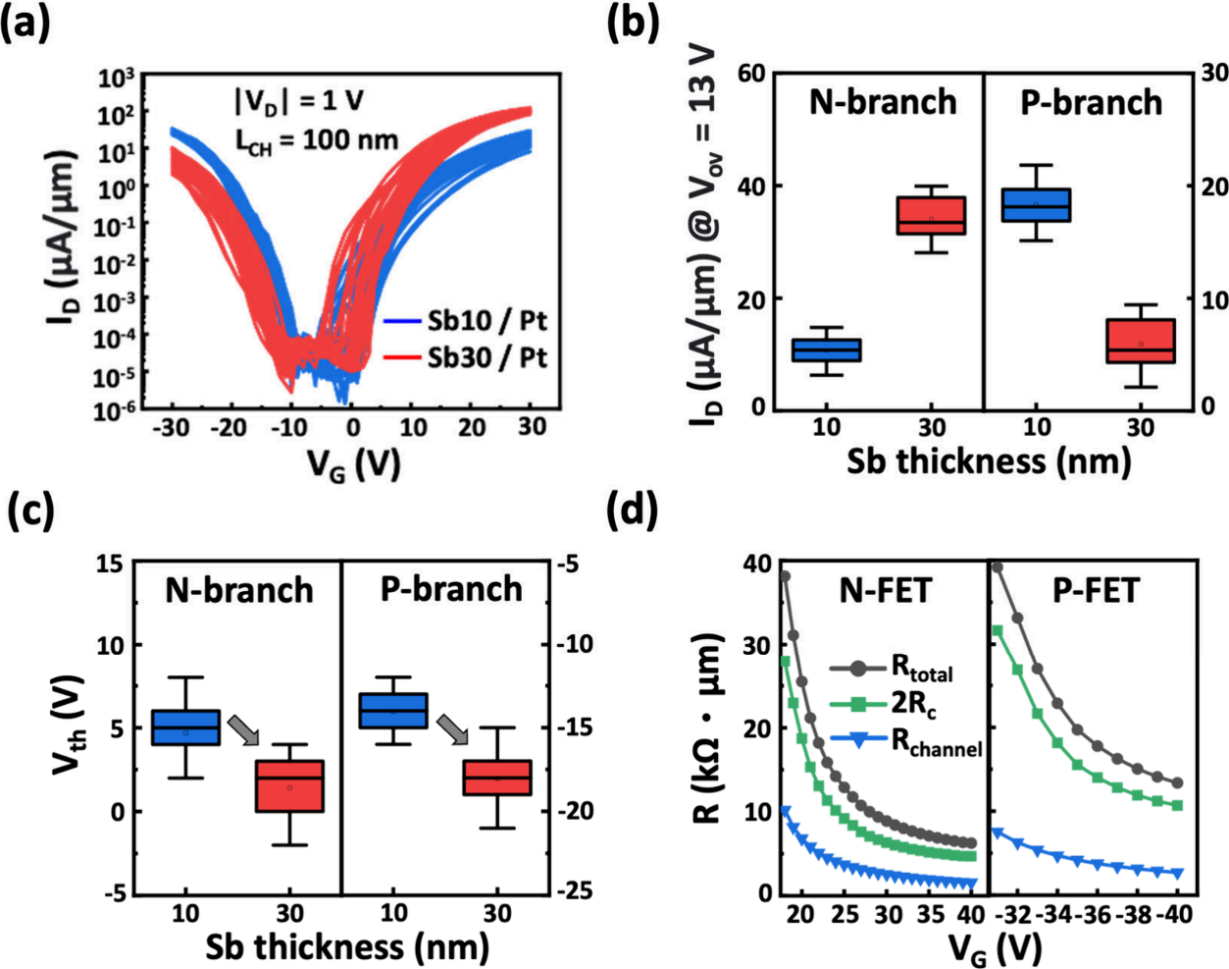
Statistical analysis of 1L-WSe_2_ FETs with different Sb/Pt contacts over 100 devices. (a) Transfer characteristics of devices with 30 nm Sb/Pt (red curves) and 10 nm Sb/Pt (blue curves) contacts. (b) On-currents at fixed overdrive voltage as extracted from data in Figure 2a and (c) V_th_ of n-/p-branch in devices with 10 and 30 nm Sb contact splits. (d) Resistance partition of n-branch and p-branch performance in devices with 30 nm-Sb/Pt contacts and 10 nm-Sb/Pt contacts, respectively.

We thus investigate the band alignment of different Sb–Pt contacts to 1L-WSe_2_ through UPS measurement to elucidate the contact type. Figure 3a illustrates the sample preparation and measurement setup in acquiring the UPS results. Sb and Pt metals are deposited onto 1L-WSe_2_ grown on a sapphire. Silver paste is applied to the Pt metal and subsequently bonded to a heavily doped silicon substrate to reduce surface charging in measurement. Prior to analysis, the sapphire substrate is removed from the sample, and the sample is immediately loaded into the characterization chamber to maintain a clean surface. Note that the entire bonding procedure with silver paste is also performed under a controlled atmosphere to eliminate interference from moisture and ensure stable vacuum conditions. As the UPS spectra display in Figure 3b, the position of the valence band maximum (VBM) in WSe_2_ in contact with metal is derived from the slope of the onset region in the spectra, with reference to the E_F_ as the zero-energy point. The values of the E_F_ – VBM difference are extracted as 0.97, 1.04, and 1.20 eV in WSe_2_ after the depositions of the 10 nm-Sb/Pt, 25 nm Pd, and 30 nm-Sb/Pt contacts, respectively. Furthermore, as derived from the secondary electron cutoff spectra at the left side of Figure 3b, the effective work functions of the Sb10/Pt, Pd, and Sb30/Pt contacts are 4.43, 4.35, and 4.19 eV at the WSe_2_ interface, respectively. The resultant energy band alignment of these contact schemes on 1L-WSe_2_ is summarized in Figure 3c. As compared to the Pd contact, the Sb–Pt bilayer contact enables modulation of the effective work function from 4.43 to 4.19 eV, thereby changing the Schottky barrier for electron and hole transport. The trend in the metal–WSe_2_ band alignment is consistent with the transition of major carrier polarity in electrical characteristics.

**Figure 3:**
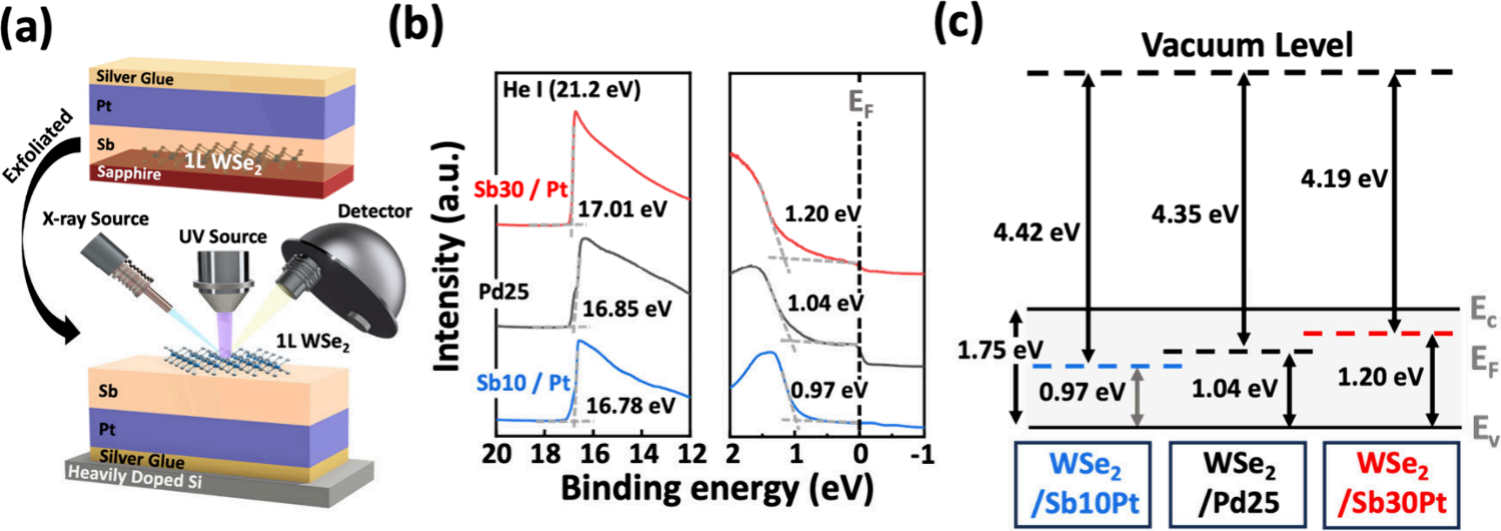
UPS analyses of 1L-WSe_2_ with Sb/Pt modulated contacts. (a) Schematic of the sample preparation and measurement setup. (b) Onset and cutoff region delineations for Pd (black curve), 10 nm-Sb/Pt (blue curve), and 30 nm-Sb/Pt (red curve) contacts. (c) Band alignment of the WSe_2_ interface with these three contact schemes.

To gain insight into the chemical interactions involved, we studied the contact and WSe_2_ interface by XPS analysis. Figure 4 displays the Sb *3d* and W *4f* core level spectra of 1L-WSe_2_ after the deposition of 10 nm-Sb/Pt and 30 nm-Sb/Pt bilayers, corresponding to the p-type and n-type contact characteristics, respectively. In the case of the 10 nm Sb layer, the Sb *3d* spectrum reveals only two pronounced peaks at 537.63 and 528.25 eV, corresponding to the Sb *3d*_*3/2*_ and Sb *3d*_*5/2*_ states of semimetallic Sb, respectively. The W *4f* spectrum exhibits two primary peaks at 34.68 and 32.50 eV, indicative of the W *4f*_*5/2*_ and W *4f*_*7/2*_ states due to the W–Se bond of WSe_2_. Additional peaks at 33.36 (Sb *4d*_*3/2*_) and 32.15 eV (Sb *4d*_*5/2*_) are reasonably attributed to the Sb–Sb bond, because they overlap energetically with the Sb *4d* spectrum.

**Figure 4:**
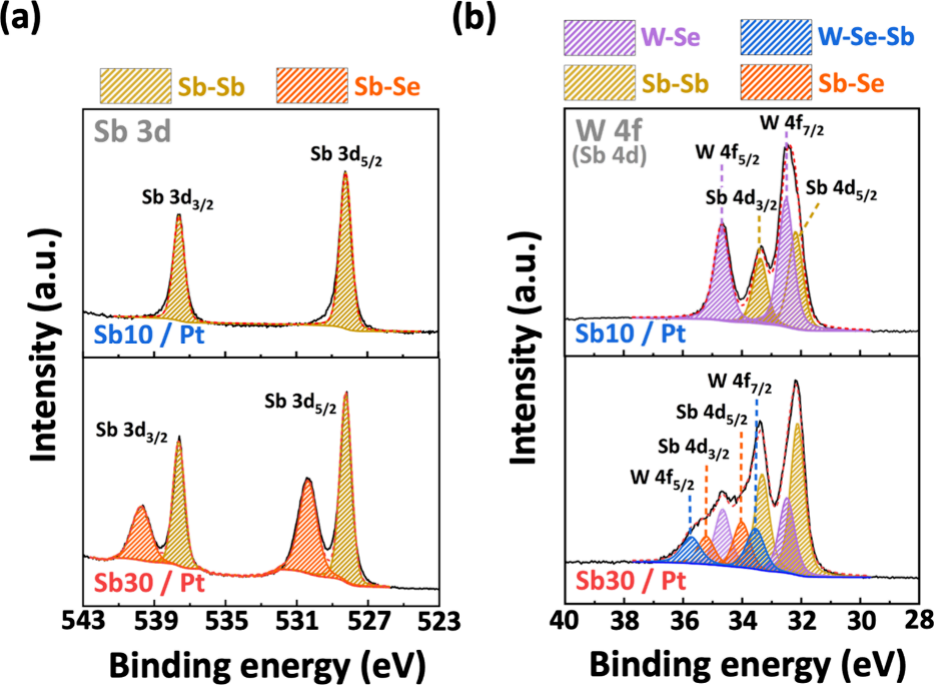
XPS analysis of the 1L-WSe_2_ film with Sb/Pt modulated contacts. Sb *3d* and W *4f* core level spectra of 10 nm-Sb/Pt and 30 nm-Sb/Pt contacts on WSe_2_. The deconvolution of chemical components is also included.

As we increase the Sb layer thickness to 30 nm, a remarkable finding in the Sb *3d* spectrum is the presence of two additional peaks at higher binding energies, specifically at 539.58 eV (Sb *3d*_*3/2*_) and 530.2 eV (Sb *3d*_*5/2*_). This observation aligns with the known characteristics of the Sb–Se bond reported in the literature.^33,34^ The Sb–Se chemical bond is also identified at 35.22 eV (Sb *4d*_*3/2*_) and 34.02 eV (Sb *4d*_*5/2*_) in the Sb *4d* (W *4f*) spectrum. Importantly, we identify another chemical component at even higher binding energies of 35.73 (W *4f*_*5/2*_) and 33.55 eV (W *4f*_*7/2*_), which is attributed to surface Sb bonding to WSe_2_, *i.e*., the W–Se–Sb bond. The formation of Se–Sb related bonds at a higher binding energy suggests a pronounced n-doping effect from the thick Sb layer on the underlying WSe_2_ channel, leading to the band realignment and thus a lower effective work function. Further, through the X-ray diffraction (XRD) analysis in Figure S2, in the 10 nm-Sb/Pt case still the amorphous film is characterized, while the 30 nm-Sb/Pt bilayer has a crystalline structure. We infer that a thin Sb amorphous layer tends to intermix with high melting point Pt metal. On the contrary, a thick Sb layer because of having a crystalline phase is relatively stable to mitigate the metal intermixing and prompt the Sb–Se bond formation.

To validate the above analysis, we perform the first-principles calculation to study the electronic structure of hexagonal-Sb on 1L-WSe_2_, based on DFT^35,36^ using the Vienna *ab initio* simulation package (VASP).^37,38^ The location of the conduction band can be understood through the projected band structure of Sb/WSe_2_ on the d_*z*_ orbital states. As shown in Figure S3, when Sb is getting close to the WSe_2_ interface, the hybridization of orbitals coupling would occur at the bonded interface. This induces the charge transfer phenomenon and actuates the electron Schottky barrier reduction from 0.59 to 0.45 eV. It is concluded that modulation of the Sb–Pt contact exhibits different chemical reactions at the WSe_2_ contact interface, thereby changing the effective contact work function as well as the polarities of the majority of the carriers. Since the Schottky contact still dominates the electrical properties, the formed Sb–Pt contact determines the V_th_ shift and on-state performance of WSe_2_ transistors.

Furthermore, we enhanced the unipolar characteristics and performance of WSe_2_ devices by implementing two different types of oxide capping layers: MoO_*x*_ for p-FETs and SiO_*x*_ for n-FETs, as the transfer characteristics displayed in Figure 5a. The deposition of the MoO_*x*_ capping layer was completed using a thermal evaporator, while the SiO_*x*_ layer was grown by using a plasma-enhanced chemical vapor deposition (PECVD) system. The on-current in p-FET with the MoO_*x*_ p-doping layer shows an increase from 66 to 170 μA/μm, while the on-current in n-FET with the SiO_*x*_ n-doping layer can improve from 137 to 165 μA/μm at |V_D_| = 1 V. Consequently, the symmetrical output characteristics are demonstrated in the Sb/Pt contact modulated WSe_2_ n-/p-FETs with oxide encapsulation (Figure 5b). The underlying doping mechanism could be explained in terms of the schematic band diagram of the 1L-WSe_2_ channel capped with respective MoO_*x*_ and SiO_*x*_ layers, as illustrated in Figure 5c,d, respectively. Given the high electron affinity of MoO_*x*_ ranging from 5.2 to 6.7 eV,^39−42^ an E_F_ realignment after contact is found to induce a large hole doping in the 1L-WSe_2_ layer.^42^ A different doping mechanism is presented when 1L-WSe_2_ was capped with SiO_*x*_; the deep donor states, arisen from oxygen vacancies, existing in amorphous SiO_*x*_ would induce n-doping in WSe_2_.^43^ We infer that the oxide encapsulation on WSe_2_ devices further aids in narrowing the Schottky barrier width in contact, resulting in a higher carrier injection probability. Accordingly, a larger driving on-current is achieved in n-/p-FETs with large on/off ratio and low hysteresis (shown in Figure S4).

**Figure 5:**
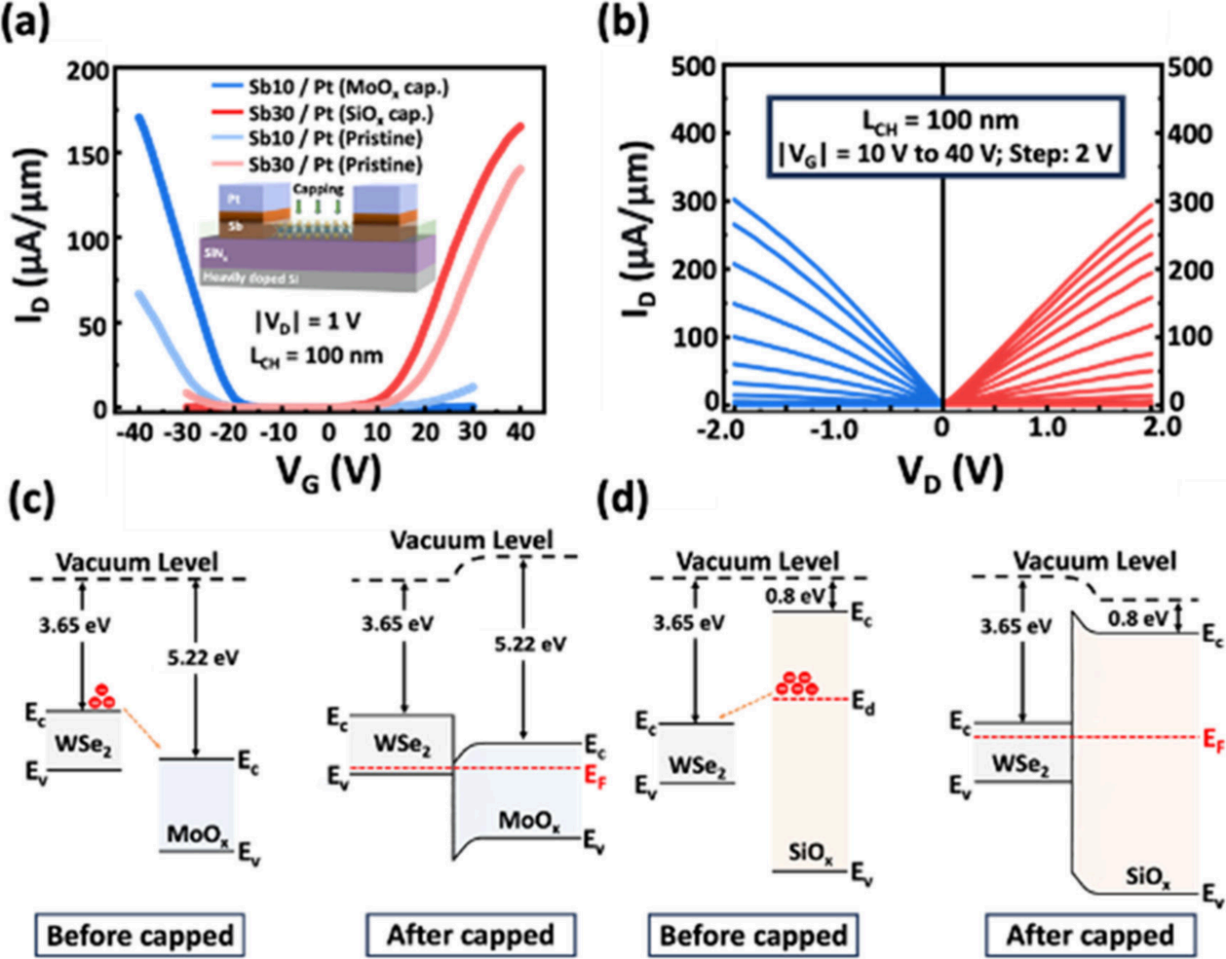
(a) Transfer characteristics of 1L-WSe_2_ FETs on the Si/SiN_*x*_ substrate with Sb/Pt modulated contact before/after the MoO_*x*_ (light blue/blue) and SiO_*x*_ (light red/red) encapsulation process. (b) Output characteristics of the Sb/Pt-contacted 1L-WSe_2_ FETs after MoO_*x*_ (blue) and SiO_*x*_ (red) encapsulation. Schematic band diagram of 1L-WSe_2_ before and after the (c) MoO_*x*_ and (d) SiO_*x*_ encapsulation.

In summary, we successfully demonstrated 1L-WSe_2_ enhanced-mode devices with selective NMOS/PMOS operation through modulation of the Sb–Pt metal contacts. The maximum on-state current reach 165 and 170 μA/μm in the separate n-FET and p-FET, respectively. The dominant carrier transport in WSe_2_ devices can be correlated to the changes in the effective interface work function due to the formation of the Sb–Se bond. We consider that the Sb–Pt modulated contact technology is promising for the application of 2D material CMOS logic electronics.

## Supporting Information Available

Characteristics of monolayer WSe_2_ FETs on the Si/SiN_*x*_ substrate with Sb/Pt contacts: device schematic, OM image, SEM image, and AFM data; XRD patterns of 10 and 30 nm Sb/Pt films on Si/SiNx substrate; DFT calculations of Sb on monolayer WSe_2_ (with and without bonded interface); and transfer characteristics of monolayer WSe_2_ FETs with Sb/Pt contacts after the oxide encapsulation logarithmic scale (PDF)
