# Supplementary material for: Antimony–Platinum Modulated Contact Enabling Majority Carrier Polarity Selection on a Monolayer Tungsten Diselenide Channel
Source: Nano Lett. 2024 Jul 9;24(29):8880–6. doi: 10.1021/acs.nanolett.4c01436 (PMC11273612; doi:10.1021/acs.nanolett.4c01436)
Supplement: Supplementary file 1 — nl4c01436_si_001.pdf [file nl4c01436_si_001.pdf]

Supporting information

Antimony-Platinum Modulated Contact Enabling Majority Carrier Polarity Selection on Monolayer Tungsten Diselenide Channel

Yu-Tung Lin,[*,§,¶] Ching-Hao Hsu,[§,¶] Ang-Sheng Chou,[¶] Zi-Yun Fong,[§] Chih-Piao Chuu,[¶] Shu-Jui Chang,[¶] Yu-Wei Hsu,[§] Sui-An Chou,[¶] San-Lin Liew,[†] Ting-Ying Chiu,[§] Fa-Rong Hou,[§] I-Chi Ni,[§] Duen-Huei Hou,[†] Chao-Ching Cheng,[¶] Iuliana P. Radu,[*,¶] and Chih-I Wu[*,§]*

[§]Graduate Institute of Photonics and Optoelectronics, National Taiwan University, Taipei 106, Taiwan

[¶]Corporate Research, Taiwan Semiconductor Manufacturing Company, Hsinchu 30091, Taiwan

[†]Quality & Reliability, Taiwan Semiconductor Manufacturing Company, Hsinchu 30091, Taiwan

Corresponding Authors

Yu-Tung Lin — Email: F09941096@ntu.edu.tw

Iuliana P. Radu — Email: iradu@tsmc.com

Chih-I Wu — Email: chihiwu@ntu.edu.tw

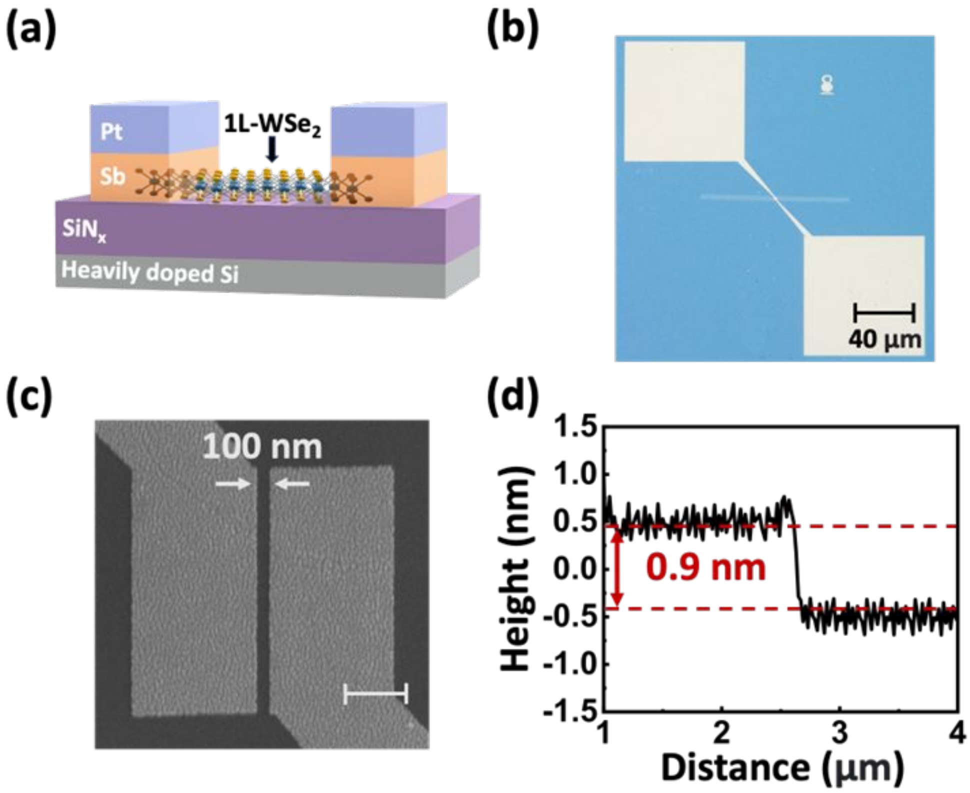

Figure S1. Characteristics of 1L-WSe_2 FETs on Si/SiN_x substrate with Sb/Pt modulated contact. (a) Device schematic. (b) Optical microscope image of a WSe_2 FET after fabrication. (c) The AFM data shows that the channel is monolayer materials. (d) SEM image demonstrates a device length of 100 nm and width of 2 µm.

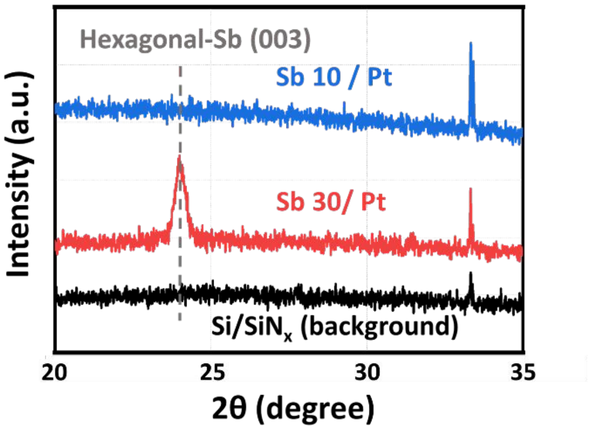

Figure S2. Analysis of X-ray diffraction patterns obtained from 10 nm and 30 nm-Sb/ Pt metal films deposited on a Si/SiN_x substrate.

[DFT method]

Here we performed the first-principles calculation to study the electronic structure of α-Sb (hexagonal) on monolayer WSe_2, based on density functional theory[1,2] using the Vienna *ab initio* simulation package (VASP).[3,4] A nearly strain-free (0.22%) interface of a supercell $\sqrt{7}$x $\sqrt{7}$ WSe_2 on 2x2 α-Sb of ~1.5 nm was constructed in a slab model with the fixed slab thickness of 55 Å and vacuum thickness of > 30 Å to eliminate the spurious interaction. The plane-wave energy cut-off was at least 550 eV. For structural optimization, all atoms were relaxed until the changes of the energy and the force reached to 10^{-5} eV and 10^{-2} eV/Å, respectively. The lattice constant of the WSe_2 and the α-Sb (hexagonal) was 3.30 Å and 4.36 Å, as determined by rev-vdW-DF2 exchange functionals with *vdW* interaction included.[5,6] Moreover, consider the optimized interlayer distance of 3.4 Å at the non-bonded Sb-WSe_2 interface, a 2.5 Å interlayer

distance was assigned to the bonded structure to investigate the effect of orbital hybridization on the Schottky barrier.

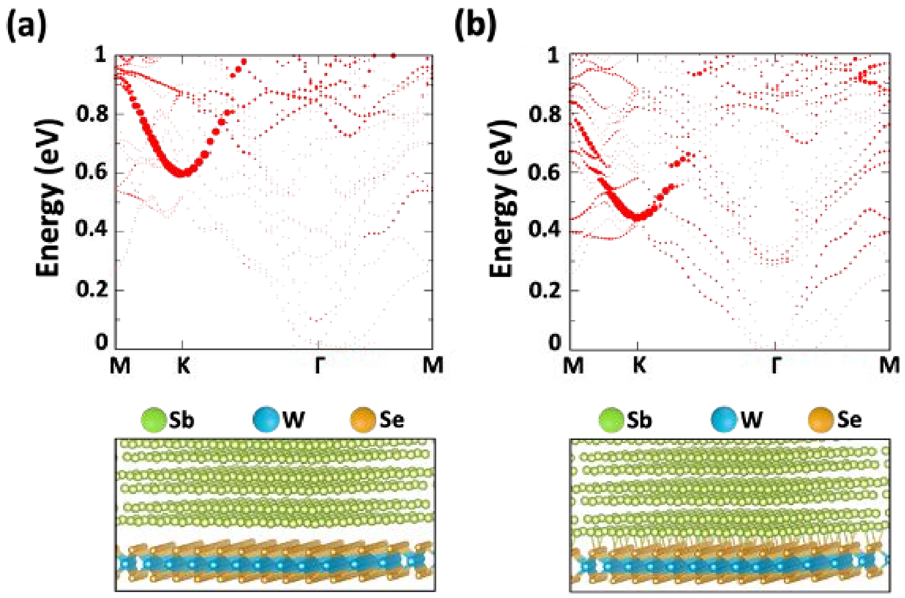

Figure S3. Projected band-structure of Sb/WSe_2 on d_z-orbital states (red circles) for (a) non-bonded interface and (b) bonded interface through the DFT calculation.

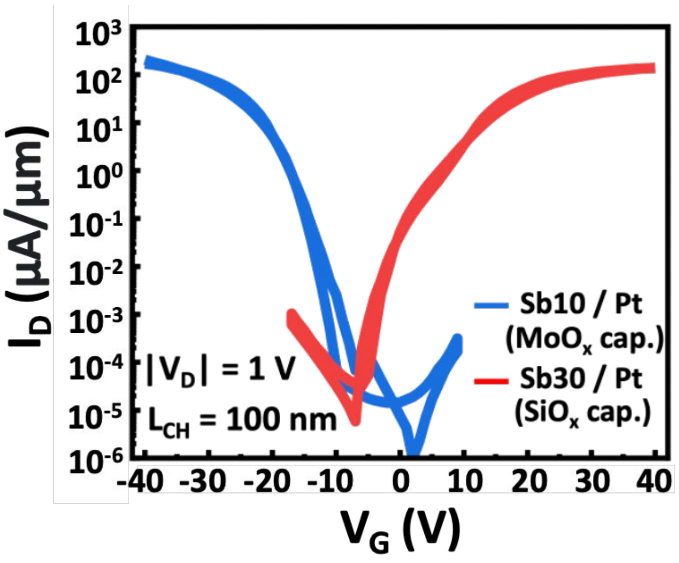

Figure S4. Transfer characteristics of 1L-WSe_2 FETs on Si/SiN_x substrate with Sb/Pt modulated contact after MoO_x and SiO_x encapsulation process under logarithmic scale.

Reference

(1) Hohenberg, P.; Kohn, W. Inhomogeneous Electron Gas. *Physical Review* 1964, *136.* (3B), B864–B871.

https://doi.org/10.1103/PhysRev.136.B864.

(1) Kohn, W.; Sham, L. J. Self-Consistent Equations Including Exchange and Correlation Effects. *Physical Review* 1965, *140* (4A), A1133–A1138. https://doi.org/10.1103/PhysRev.140.A1133.

(3) Kresse, G.; Furthmüller, J. Efficiency of Ab-Initio Total Energy Calculations for Metals and Semiconductors Using a Plane-Wave Basis Set. *Computational Materials Science* 1996, *6* (1), 15–50.

https://doi.org/10.1016/0927-0256(96)00008-0.

(4). Kresse, G.; Furthmüller, J. Efficient Iterative Schemes for *Ab Initio* Total-Energy Calculations Using a Plane-Wave Basis Set. *Physical Review B* 1996, *54* (16), 11169–11186.

https://doi.org/10.1103/PhysRevB.54.11169.

(5) Hamada, I. Van Der Waals Density Functional Made Accurate. *Physical Review B* 2014, *89* (12), 121103.

https://doi.org/10.1103/PhysRevB.89.121103.

(6) Hamada, I. Erratum: Van Der Waals Density Functional Made Accurate. *Physical Review B* 2015, *91* (11), 119902.

https://doi.org/10.1103/PhysRevB.91.119902.
